# Large-scale genomic analysis reveals the genetic cost of chicken domestication

**DOI:** 10.1186/s12915-021-01052-x

**Published:** 2021-06-16

**Authors:** Ming-Shan Wang, Jin-Jin Zhang, Xing Guo, Ming Li, Rachel Meyer, Hidayat Ashari, Zhu-Qing Zheng, Sheng Wang, Min-Sheng Peng, Yu Jiang, Mukesh Thakur, Chatmongkon Suwannapoom, Ali Esmailizadeh, Nalini Yasoda Hirimuthugoda, Moch Syamsul Arifin Zein, Szilvia Kusza, Hamed Kharrati-Koopaee, Lin Zeng, Yun-Mei Wang, Ting-Ting Yin, Min-Min Yang, Ming-Li Li, Xue-Mei Lu, Emiliano Lasagna, Simone Ceccobelli, Humpita Gamaralalage Thilini Nisanka Gunwardana, Thilina Madusanka Senasig, Shao-Hong Feng, Hao Zhang, Abul Kashem Fazlul Haque Bhuiyan, Muhammad Sajjad Khan, Gamamada Liyanage Lalanie Pradeepa Silva, Le Thi Thuy, Okeyo A. Mwai, Mohamed Nawaz Mohamed Ibrahim, Guojie Zhang, Kai-Xing Qu, Olivier Hanotte, Beth Shapiro, Mirte Bosse, Dong-Dong Wu, Jian-Lin Han, Ya-Ping Zhang

**Affiliations:** 1 State Key Laboratory of Genetic Resources and Evolution, Yunnan Laboratory of Molecular Biology of Domestic Animals, Kunming Institute of Zoology, Chinese Academy of Sciences, Kunming, 650223 China; 2 Kunming College of Life Science, University of Chinese Academy of Sciences, Kunming, 650204 China; 3 Howard Hughes Medical Institute, University of California Santa Cruz, Santa Cruz, CA 95064 USA; 4 Department of Ecology and Evolutionary Biology, University of California Santa Cruz, Santa Cruz, CA 95064 USA; 5 College of Animal Science and Technology, Anhui Agricultural University, Hefei, 230036 China; 6 Key Laboratory of Animal Genetics, Breeding and Reproduction of Shaanxi Province, College of Animal Science and Technology, Northwest A&F University, Yangling, 712100 China; 7 Museum Zoologicum Bogoriense, Research Center for Biology, Indonesian Institute of Science (LIPI), Cibinong, Bogor, 16911 Indonesia; 8 CAAS-ILRI Joint Laboratory on Livestock and Forage Genetic Resources, Institute of Animal Science, Chinese Academy of Agricultural Sciences (CAAS), Beijing, 100193 China; 9 Key Laboratory of Agricultural Animal Genetics, Breeding and Reproduction, The Cooperative Innovation Center for Sustainable Pig Production, Ministry of Education, Huazhong Agricultural University, Wuhan, 430070 China; 10 Zoological Survey of India, New Alipore, Kolkata, West Bengal 700053 India; 11 School of Agriculture and Natural Resources, University of Phayao, Phayao, 56000 Thailand; 12 Unit of Excellence on Biodiversity and Natural Resources Management, University of Phayao, Phayao, 56000 Thailand; 13 Department of Animal Science, Shahid Bahonar University of Kerman, P.O. Box 76169133, Kerman, Iran; 14 Faculty of Agriculture, University of Ruhuna, Matara, Sri Lanka; 15 Institute of Animal Husbandry, Biotechnology and Nature Conservation, University of Debrecen, Debrecen, H-4032 Hungary; 16 Institute of Biotechnology, School of Agriculture, Shiraz University, P.O. Box 1585, Shiraz, Iran; 17 Center for Neurobiology and Brain Restoration, Skolkovo Institute of Science and Technology, Moscow, 143026 Russia; 18 Center for Excellence in Animal Evolution and Genetics, Chinese Academy of Sciences, Kunming, 650204 China; 19 Dipartimento di Scienze Agrarie, Alimentarie Ambientali, University of Perugia, 06123 Perugia, Italy; 20 BGI-Shenzhen, Beishan Industrial Zone, Shenzhen, 518083 China; 21 Laboratory of Animal Genetics, Breeding and Reproduction, National Engineering Laboratory for Animal Breeding, College of Animal Science and Technology, China Agricultural University, Ministry of Agriculture of China, Beijing, 100193 China; 22 Bangladesh Agricultural University, Mymensingh, 2202 Bangladesh; 23 Cholistan University of Veterinary and Animal Sciences, Bahawalpur, Pakistan; 24 Department of Animal Science, University of Peradeniya, Peradeniya, 20400 Sri Lanka; 25 National Institute of Animal Husbandry, Hanoi, Vietnam; 26 Livestock Genetics Program, International Livestock Research Institute (ILRI), Nairobi, 00100 Kenya; 27 China National Genebank, BGI-Shenzhen, Shenzhen, 518083 China; 28 Centre for Social Evolution, Department of Biology, University of Copenhagen, DK-1870 Copenhagen, Denmark; 29 Yunnan Academy of Grassland and Animal Science, Kunming, 650212 China; 30 Cells, Organisms and Molecular Genetics, School of Life Sciences, University of Nottingham, Nottingham, NG7 2RD UK; 31 Livestock Genetics Program, International Livestock Research Institute (ILRI), P.O. Box 5689, Addis Ababa, Ethiopia; 32 Wageningen University & Research - Animal Breeding and Genomics, 6708 PB Wageningen, The Netherlands; 33 State Key Laboratory for Conservation and Utilization of Bio-resource, Yunnan University, Kunming, 650091 China

**Keywords:** Domestication, Bottleneck, Genetic load, Deleterious mutation, Domestic chicken

## Abstract

**Background:**

Species domestication is generally characterized by the exploitation of high-impact mutations through processes that involve complex shifting demographics of domesticated species. These include not only inbreeding and artificial selection that may lead to the emergence of evolutionary bottlenecks, but also post-divergence gene flow and introgression. Although domestication potentially affects the occurrence of both desired and undesired mutations, the way wild relatives of domesticated species evolve and how expensive the genetic cost underlying domestication is remain poorly understood. Here, we investigated the demographic history and genetic load of chicken domestication.

**Results:**

We analyzed a dataset comprising over 800 whole genomes from both indigenous chickens and wild jungle fowls. We show that despite having a higher genetic diversity than their wild counterparts (average π, 0.00326 vs. 0.00316), the red jungle fowls, the present-day domestic chickens experienced a dramatic population size decline during their early domestication. Our analyses suggest that the concomitant bottleneck induced 2.95% more deleterious mutations across chicken genomes compared with red jungle fowls, supporting the “cost of domestication” hypothesis. Particularly, we find that 62.4% of deleterious SNPs in domestic chickens are maintained in heterozygous states and masked as recessive alleles, challenging the power of modern breeding programs to effectively eliminate these genetic loads. Finally, we suggest that positive selection decreases the incidence but increases the frequency of deleterious SNPs in domestic chicken genomes.

**Conclusion:**

This study reveals a new landscape of demographic history and genomic changes associated with chicken domestication and provides insight into the evolutionary genomic profiles of domesticated animals managed under modern human selection.

**Supplementary Information:**

The online version contains supplementary material available at 10.1186/s12915-021-01052-x.

## Background

All organisms carry a certain level of deleterious mutations in their genomes, which can potentially affect their fitness [1, 2]. The majority of these harmful mutations are detrimental and recessive—only a few are dominant or recessive lethal [3]. Such mutations can best be seen as high-impact mutations (that is, affecting the functioning or expression of a gene) [4, 5]. Most of them have a negative effect; however, some may result in a desirable phenotype and are therefore maintained by natural and artificial selection. Some of these alleles that are preferred in an artificial breeding setting would nevertheless be detrimental in the wild. The evolution of domestic species is characterized by the exploitation of high-impact mutations during inbreeding, artificial selection, and post-divergence gene flow [6–10], which could affect the occurrence of both desired and undesired high-impact mutations [11]. Extensive studies have reported that domestic species, such as horses [12], dogs [13], rice [14, 15], sheep [10], and tomatoes [16], are burdened by many more deleterious mutations than their wild relatives. The “cost of domestication” hypothesis was proposed to explain this general pattern observed in these domestic species [17]. It suggests that bottlenecks along with domestication reduced the power of purifying selection to remove deleterious variants, therefore resulting in a dramatic accumulation of deleterious variants in domesticated species. However, this model cannot be generalized to all major domesticates because some of them lack a domestication bottleneck [18, 19]. For example, genomic assessment of pigs [20], bees [21], and some crops [18] exhibited no significant historical decline in their genetic diversity relative to respective wild progenitors.

Chicken is believed to have been domesticated from the red jungle fowl (RJF). Early genomic studies have identified a number of variants that differentiate chicken from RJF, facilitating our understanding of the genetic changes underlying chicken domestication [22, 23]. It was thought that chicken has been domesticated via a commensal pathway within the Holocene, in which its early domestication was assumed to be fully unintentional [8] and not marked by a bottleneck [19]. However, some studies proposed a contradictory opinion based on the observations of evaluated high-impact mutations in specific chicken breeds [2, 24], although the establishment of these fancy and commercial birds has likely resulted in a more recent bottleneck. To date, whether there was a domestication bottleneck, its strength and effect, and how it potentially shaped the pattern of high-impact mutations in relation to other factors, including admixture and selection, are unresolved in domestic chicken.

Measuring the magnitude of domestication bottleneck by direct comparison of genetic diversity in current domesticates with their true wild progenitors is often impossible (e.g., extinction of the ancestors of cattle and horses) or complicated by several factors [8, 25, 26]. First, accurate identification of the sole ancestor(s) of domesticated species remains very challenging because the potential wild relatives either continue to evolve as closely related genetic entities with a large geographic range (e.g., RJFs and gray wolves) [27–29] or survive only in very small fragmented populations (e.g., sheep, goats, and buffaloes) [30, 31] due to overhunting among other human activities. Second, hybridization between domesticated and wild populations is common in nature and thus could mislead the estimation of their genetic differences [32, 33]. Domesticated chickens have been affected by gene flow via hybridization with other RJFs and jungle fowl species over thousands of years [34, 35]. RJFs are widely distributed and could be assigned into five subspecies (*G. g. spadiceus*, *G. g. murghi*, *G. g. jabouillei*, *G. g. gallus*, and *G. g. bankiva*) ranging across South and Southeast Asia where they may have been hybridizing with village hens [36]. The third is the aforementioned issue that wild species can rarely escape from climate and anthropogenic pressures, and this creates somewhat parallel trajectories to evolution under domestication [28, 37].

In the first phase of our 1K Chicken Genomes Project (1K CGP; https://bigd.big.ac.cn/chickensd/), through sequencing and analyzing 863 genomes from both jungle fowls and indigenous chickens sampled across South, Southeast, and East Asia as well as Europe (including 149 RJFs covering all five subspecies sampled in their natural ranges), we demonstrated that all domestic chickens were monophyletic, derived from an RJF lineage of *G.* g. *spadiceus* (GGS) whose present-day range is predominantly in northern Thailand, southwestern China, and Myanmar, and then regained genetic diversity via introgression from additional RJF subspecies and jungle fowl species during their dispersals out of the domestication center [27]. In this study, based on the new knowledge and genomes (including 696 domestic chickens spanning Eurasia and 45 GGS from Thailand and Yunnan Province, China), we examined the demographic history for chickens before and after their domestication, investigated the distribution and frequency of high-impact mutations across their genomes, and estimated the genetic cost of chicken domestication and breed formation.

## Results

### Evidence of bottleneck in chicken domestication

**Fig. 1:**
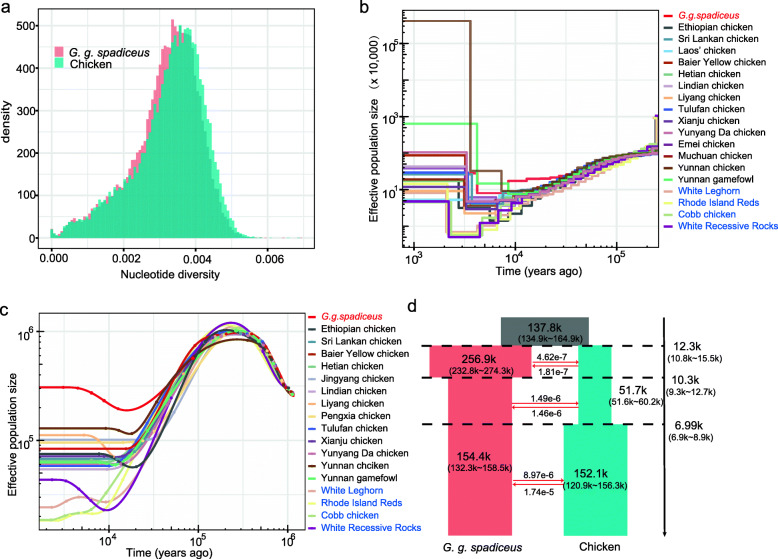
Genomic diversity and demographic history for both domestic chicken and *G. g. spadiceus*. **a** Nucleotide diversity for domestic chicken and *G. g. spadiceus*. In this analysis, the average nucleotide diversity for domestic chicken was calculated based on 696 samples, and for *G. g. spadiceus*, it was calculated based on 35 samples (after removing 10 admixed samples). **b** MSMC analysis of the historical population size of 18 chicken populations and GGS. **c** SMC++ analysis of the historical population size of 17 chicken populations and GGS. A bottleneck is evident in all chicken populations and pronounced in commercial chickens. Breed information for commercial chickens was in blue. **d** *Dadi* analysis showing the divergence and splitting of domestic chickens from GGS

To have baseline information on the genomic diversity for present-day domestic chickens and GGS, we estimated nucleotide diversity (π) for each population. The average π for all domestic chickens was 3.26e−3, slightly higher than that for GGS (mean 3.16e−3; *P* < 2.2e−16, Wilcoxon signed-rank test; Fig. 1a). We note that this result should be interpreted with caution because our sampling of GGS likely did not cover all their genetic diversity while our chicken samples were from a wide range sampling spanning Eurasia. Pooling analysis of genomes from these diverse chicken populations together possibly inflates the estimation of genetic diversity (Additional file 1: Figure S1).

We used the pair-wise sequential Markovian coalescent (PSMC) [38] approach to estimate the historical effective population sizes (Ne) of domestic chickens and GGS. As the efficiency of this analysis relies on heterozygotes across genomes [39], the result from low-coverage genomes is unreliable. The number of high-coverage genomes in 1K CGP is limited [27], so we also included recently published chicken genomes [35, 40, 41] with a coverage of ≥ 20-folds in this analysis (Additional file 1: Table S1). Our data contain genomes from GGS and 18 chicken populations including commercial breeds (White Leghorn, White Recessive Rocks, Rhode Island Red, and Cobb chicken), Ethiopian, Sri Lankan, Laos, and Chinese local chickens. The estimations were scaled by a mutation rate of 1.91e−9 substitution per site per year and a generation time of 1 year [42]. PSMC revealed that both domestic chickens and GGS had nearly identical demographic histories before 20 thousand years ago (kya) (Additional file 1: Figure S2), which is expected as the chicken was originated from GGS [27]. Specifically, initialed at 1 million years ago (Mya), Ne for chicken and GGS showed expansion and reached a maximum of around 100 kya, followed by continuous contraction until 20 kya. The cycles of past population expansions and contractions were similar to those observed in other wild birds, supporting the claim that climate fluctuations during the Quaternary have likely shaped the evolution and speciation of many bird species [43].

However, PSMC has limited power to reveal the recent Ne within 10 kya. We then used MSMC [44], a method similar to PSMC, which could analyze genomes from more than one individual for each population and therefore has an enhanced power to infer the demographic changes for relatively recent evolutionary events like domestication. Our estimations were based on four haplotype genomes (two individuals) for each population. Consistent with PSMC, MSMC revealed a comparable and dramatic Ne contraction for 18 chicken populations and GGS between 100 and 20 kya (Fig. 1b). Thereafter, chicken and GGS showed obvious differentiation. Specifically, Ne for GGS remained relatively constant before the rapid decline onset ~8 kya but then stabilized ~4 kya, while domestic chickens showed a continuous Ne decline until Yunnan local chicken and Yunnan game fowl recovered ~6–7 kya and other chicken populations recovered later ~2–4 kya. Commercial chicken breeds, including White Recessive Rocks, Cobb chicken, White Leghorn, and Rhode Island Red, have much smaller Ne than other chickens, and their Ne recovered much later (~2 kya), consistent with the fact that commercial chickens have been subject to intensified artificial selection and inbreeding. It should be noted that more recent Ne estimated by MSMC tends to be inflated [44], especially in analyzing more than two haplotypes. Here, we further inferred historical Ne for GGS and each chicken population using SMC++ [45], a method estimating demographic histories based on multiple genomes without phasing. For each population, we allowed five genomes with sequencing coverage over 15-folds, yielding a total of 17 chicken populations for the analysis from our current dataset (Fig. 1c). This analysis also revealed strong evidence of domestication bottleneck for chicken compared with GGS, broadly consistent with the result from MSMC. Compared with MSMC, SMC++ has a higher resolution for estimating recent population histories. For example, SMC++ analyses also revealed that commercial chickens (White Leghorn, White Recessive Rock, Rhode Island Red, and Cobb chicken) have a stronger bottleneck and much smaller recent Ne compared with other chickens. Ne for Yunnan local chicken, Liyang chicken, White Recessive Rocks, Sri Lankan local chicken, Ethiopian local chicken, and game fowls showed a recent recovery after the bottleneck during 10 kya.

Because PSMC, MSMC, and SMC++ do not take into account the possible effects of admixture [46, 47], the estimated initiation of Ne differentiation does not necessarily correlate with the splitting time. To further infer the evolutionary history of domestic chickens, we used *dadi* [48] to fit the joint site frequency spectrum (SFS) between domestic chicken and GGS populations. We tested four assumed demographic models (Additional file 1: Figure S3-S4 and Tables S2-S3) and found that model 3 had the highest likelihood (Fig. 1d), suggesting its best fit to observed SFS. Under this model, we estimated that modern domestic chickens and GGS separated from each other ~12,300 (95% confident intervals (CI)10,800–15,500) years ago. After that, GGS showed a slight expansion in Ne until 10,300 years ago (95% CI 9300–12,700), followed by a contraction from around 257,000 down to 154,000 birds, which was likely resulted from recent habitat loss and overhunting. On the contrary, the average Ne for domestic chickens dramatically declined from around 138,000 birds on their separation from GGS down to 52,000 until ~6990 (6900–8900) years ago when they started to be recovered up to ~152,000 birds till today. MSMC, SMC++, and *dadi* analyses suggested that domestic chickens experienced a continuous Ne decline since their splitting off from GGS, indicating a bottleneck in chicken domestication. We noted that admixture with local jungle fowl or other domestic chicken populations is pervasive [27], which likely affects the inference of demographic history [49], so the bottleneck time we estimated here is inconclusive.

### Identification of high-impact mutations

**Fig. 2:**
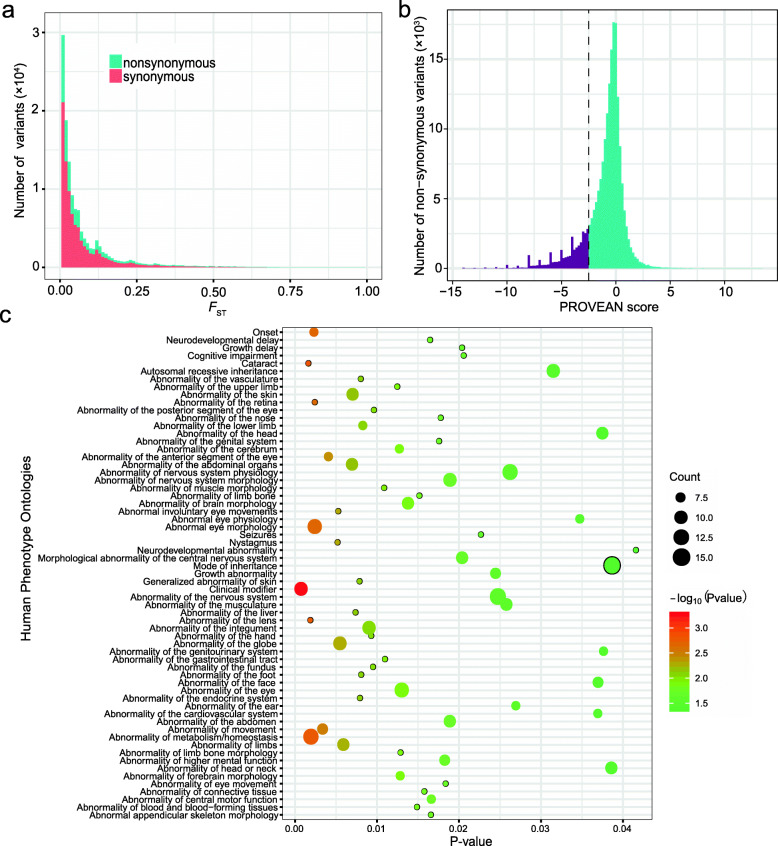
The distribution and functional enrichment analyses of high-impact mutations. **a** Distribution of pairwise *F*_ST_ between domestic chickens and GGS for non-synonymous and synonymous mutations (stacked on the plot). **b** Distribution of the effects of variants predicted by PROVEAN. The more negative the score is, the more likely the variant impacts protein function. The PROVEAN score threshold used in this study is drawn as a vertical dashed line (score ≤ −2.5). **c** HPO analysis of genes carrying alleles with PROVEAN score < −10. *P*-values were corrected using Benjamini-Hochberg FDR. Count depicts the number of genes for each category. We only show HPO terms with more than six enriched genes

To measure whether the domestication-associated bottleneck could have induced the rise of high-impact SNPs (hSNPs) in chicken genomes, we analyzed the variants called in domestic chicken and GGS genomes among our 1K CGP. We found that protein-coding regions accounted for ~4.2% of the chicken genome, and 1.6% of the genomic variants (435,919) were present in these exonic regions (Fig. 2a), which were nearly two times higher than those observed in other domestic animals including dogs, pigs, cattle, and horses (Additional file 1: Table S4). These exonic variants were only differentiated slightly between domestic chickens and GGS, suggesting that they have been subjected to evolutionary constraints. We classified mutations from the exonic regions into non-synonymous and synonymous substitutions and identified 146,193 non-synonymous SNPs, accounting for around 33.5% of the total exonic SNPs. The estimated ratio for the numbers of non-synonymous SNPs over exonic SNPs was similar to those observed in other domestic animals (Additional file 1: Table S4). To assess their potential effects (i.e., tolerant or deleterious) on associated amino acid changes in protein sequences of domestic chickens, we performed a PROVEAN [50] analysis. Based on the score threshold ≤ −2.5, we detected 22,282 potential hSNPs (Fig. 2b). Compared to mammals, bird chromosomes are highly variable in size. Chicken chromosomes are classified into three classes including 5 macrochromosomes (chrs: 1–5), 28 microchromosomes (chrs: 11–38), and 4 intermediate chromosomes (chrs: 6–10) [51]. We compared the PROVEAN scores of non-synonymous mutations and found that the average PROVEAN scores (damaging effect) of variants on the microchromosomes were significantly lower than those on the remaining chromosomes (Additional file 1: Figure S5). This finding is consistent with the previous report that microchromosomes have been subjected to evolutionary constraint or more efficient purifying selection because of their higher recombination rates [51], and therefore, mutations in conserved regions are more likely to be harmful.

To evaluate the potential biological roles of the associated genes, we retrieved genes carrying the non-synonymous SNPs of PROVEAN score < −10 and performed functional enrichment analyses including GO and Human Phenotype Ontology (HPO). A total of 346 protein-coding genes were retrieved, and they are involved in multiple functional GO and HPO categories, including abnormalities of nervous system physiology, growth, bone and muscle development and morphology, cardiovascular and respiratory system, metabolism/homeostasis, vision, and immunity (Additional file 1: Table S5, Fig. 2c). These mutations were at low frequencies in domestic chickens, most of them are likely linked to health problems faced by modern poultry industries, as highly productive birds have been suffering from brittle bones, blindness, crippling leg disorders, ascites (a disease of the lungs and heart), and sudden death syndrome [52, 53]. Nevertheless, a few of them may be the target of positive selection during domestication and/or recent genetic improvement or breeding for specific traits.

### Validation of the function of *TSHR*-Gly558Arg using a transgenic mouse model

In an early investigation [22], thyroid-stimulating hormone receptor (*TSHR*) showed the strongest signal of selection, with one missense mutation (chr5:40,089,599G/A; *TSHR*-Gly558Arg) being nearly fixed in domestic chickens compared to RJFs (< 23%; unclear subspecies classification). A recent paleogenetic study, however, showed the timing of selection on this gene in ancient and modern European chickens and concluded that the dramatic rise in frequency to modern ubiquity only began 1.1 kya [54]. Our recent study showed that this mutation was nearly fixed (> 90%) in both indigenous chickens and GGS but maintained at extremely low frequencies among other RJF subspecies [27]. Should any claims be based on the knowledge that this mutation is functional, however, the general biological role of this gene and the specific functional consequence of this mutation have not been resolved in domestic chickens. In our analysis, the *TSHR*-Gly558Arg has a PROVEAN score at −6.981, to be indicative of potentially high effect (< −2.5). Therefore, it is interesting to assess the potential biological function of this mutation as a potential proof of concept to test all our predictions.

**Fig. 3:**
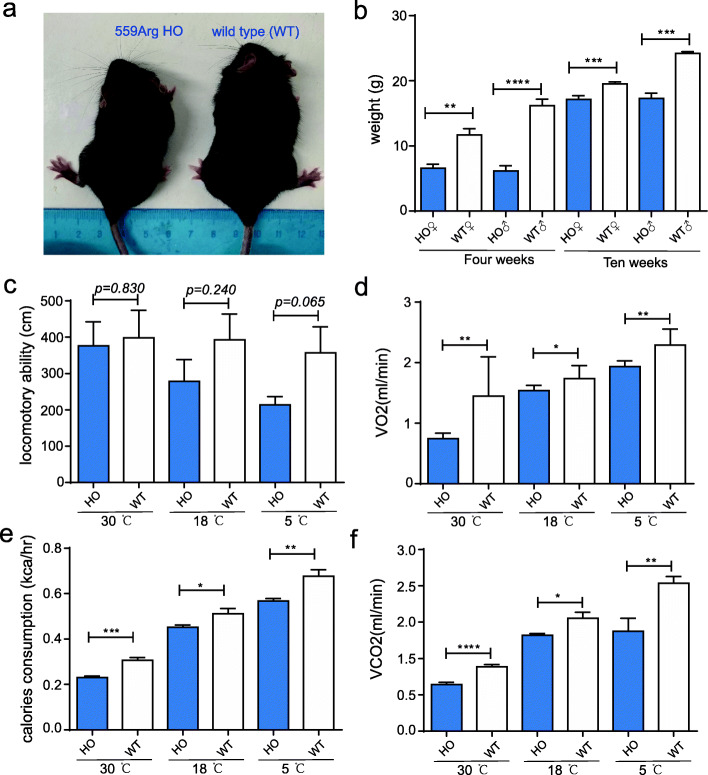
Testing the function of *TSHR*-Gly559Arg using transgenic mouse model assay. **a** Photograph showing *TSHR*-559Arg knock-in homozygous (HO) and wild-type (WT) mice at 4 months old. **b** Bar plot shows that HO mice have significantly lower body weight than wild-type mice. **c** No difference in total locomotive ability between HO and wild-type mice. **d**–**f** HO mice have significantly lower oxygen consumption (VO_2_), calorie consumption, and carbon dioxide exhalation (VCO_2_) compared to wild-type mice. In **b**, *n* = 6 HO and *n* = 8 WT female and *n* = 8 HO and *n* = 8 WT male 4-week-old mice, as well as *n* = 7 HO and *n* = 7 WT female and *n* = 12 HO and *n* = 14 WT male 10-week-old mice were analyzed. In **c**–**f**, *n* = 8 for both HO and WT male mice were analyzed for each test. *P < 0.05; **P < 0.01;***P < 0.001; ****P < 0.0001. Statistical significance was measured by Student’s t-test (two-tailed)

Because of the challenge in editing an avian genome efficiently and precisely, several studies have employed transgenic mouse or human cell lines [55] or zebrafish [56, 57] models to test the potential roles of specific mutations of interest identified in the bird genomes. As *TSHR* is associated with development and metabolism in mice [58] and glycine at this position in chicken is conserved among all known vertebrate TSHR amino acid sequences [22, 59], we constructed chicken *TSHR-*558Arg knock-in mice (matched with mice *TSHR-*559Arg) to test whether this mutation has any biological effects (Additional file 1: Figure S6 and Table S6). At normal conditions, *TSHR-*559Arg homozygous mice developed significantly smaller (*P* < 0.01) bodies in both sexes than the wild-type mice (Fig. 3a, b, Additional file 1: Figure S7). At 30 °C, 18 °C, and 5 °C experimental conditions, we measured the physical movements of the mice by counting their locomotor activities and found no significant difference (*P* > 0.05) between the *TSHR-*558Arg homozygous and wild-type mice (Fig. 3c). We also recorded their energy expenditures and metabolism rates and realized that the *TSHR-*559Arg homozygous mice had less food uptake (Additional file 1: Figure S8) and significantly lower oxygen (VO_2_), calorie consumptions, and carbon dioxide production (VCO_2_) than the wild-type mice (Fig. 3d–f; *P* < 0.05). This result suggests that *TSHR*-Gly558Arg is biologically functional in chickens. However, such validation for a single mutation cannot stand the effect of all mutations; further experimental validation for additional and specific variants is warranted.

### Increased genetic load in domestic chickens

**Fig. 4:**
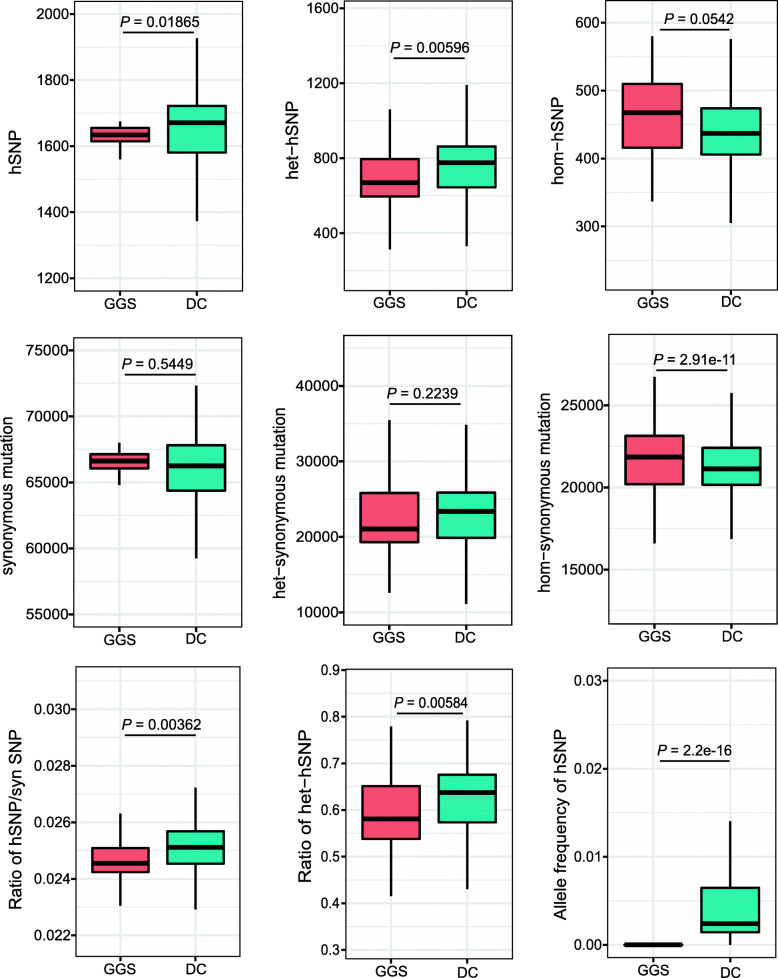
The frequency and number of high-impact mutations in domestic chickens and *G. g. spadiceus*. *P*-values were computed by the Wilcoxon signed-rank test between domestic chicken (DC; *n* = 696) and *G. g. spadiceus* (GGS; *n* = 35)

To compare the levels of genetic loads between domestic chickens and GGS, we evaluated the numbers and frequencies of non-synonymous and synonymous SNPs as well as the hSNPs among their genomes (Fig. 4). Our result showed that each chicken carried approximately 2.95% more hSNPs than GGS across their genomes (*P* = 0.01865, Wilcoxon signed-rank test; Fig. 4). Domestic chickens also had a significantly higher ratio of hSNPs relative to synonymous SNPs (*P* = 1.83e−6, Wilcoxon signed-rank test; Fig. 4). In particular, the average allele frequency of hSNPs was significantly higher in domestic chickens than in GGS (*P* < 2.2e−16, Wilcoxon signed-rank test; Fig. 4). Our previous study suggests that, after originating from GGS, domestic chickens were further admixed with other jungle fowls during their dispersal out the domestication center [27]. The magnitude of gene flow is greatest between chickens and local jungle fowls, which might bias the estimation of genetic load. We further measured the number and ratio of hSNPs in some potentially drifted and/or isolated populations with no jungle fowl distributed in their natural ranges. To maximize more samples for each population, we chose Tibetan chicken, Beijing You chicken, Silkie chicken, chickens from Xinjiang province of China, and the White Leghorn chicken for comparison. The ratio of deleterious mutation relative to synonymous mutation and the level of heterozygous deleterious mutation varied among these chicken populations but were all higher than GGS (Additional file 1: Figure S9). Collectively, these analyses suggest that domestication has led to a rapid accumulation of high-impact mutations, and thus, the genetic burden/load defined by the hSNPs was likely increased in domestic chickens.

We next compared the levels of hSNPs in both homozygous and heterozygous states and observed 62.4% hSNPs in domestic chickens to be maintained in heterozygote states, significantly higher than that in GGS (57.8%; *P* = 0.00584, Wilcoxon signed-rank test; Fig. 4). In addition, domestic chickens carried far more heterozygous hSNPs (*P* = 0.00596, Wilcoxon signed-rank test) but less homozygous hSNPs (*P* = 0.0542, Wilcoxon signed-rank test; Fig. 4) than GGS (Fig. 4). However, the number of synonymous alleles, and these in the heterozygous states per genome, was comparable between domestic chickens and GGS (*P* = 0.5449 and 0.2239, Wilcoxon signed-rank tests). The total number of homozygous synonymous alleles was higher in GGS than in domestic chickens (*P* = 2.91e−11, Wilcoxon signed-rank test; Fig. 4). Similarly, we measured the level of heterozygous hSNPs in Tibetan chicken, Beijing You chicken, Silkie chicken, chickens from Xinjiang province of China, and the White Leghorn chicken and found these populations also had a higher ratio and number of heterozygous hSNPs than GGS (Additional file 1: Figure S9). These results suggest that the heterozygous mutation load was elevated in domestic chickens.

### The pattern of high-impact mutations in selective sweeps

How does selection affect the occurrence of hSNPs in domestic chicken genomes? To explore this, we retrieved the putatively selective sweeps identified by locus-specific branch length (LSBL) and π-ratio from our previous study [27] and compared the distribution pattern of hSNPs mapped within these sweeps with that in the remaining chicken genomic regions. Each of three sets of selective sweeps defined by LSBL(chicken, GGS, *G. g. jabouillei*) or LSBL(chicken, GGS, *G. g. murghi*) or π-ratio analysis possessed lower numbers of hSNPs (Additional file 1: Figure S10). This finding mirrors the observations in cassava [60] and grape [61], suggesting that the genes under selection tend to delimit hSNPs and/or to favor haplotypes carrying fewer hSNPs following the domestication and dispersal processes [60–62]. In addition, all the selective sweeps identified by each of the three statistics showed higher frequencies of hSNPs in domestic chickens than in GGS (Additional file 1: Figure S10). Some of these hSNPs may be the targets of selection to confer advantages in phenotypic or adaptive innovations, while most of them were likely a result of hitchhiking with nearby positively selected alleles.

## Discussion

Chickens are among the few domestic species with their progenitors extant in the wild today, providing an excellent system to address questions about evolutionary changes under domestication. In this work, we conducted systematic genomic studies of the domestication history and landscape of hSNPs using the largest genomic dataset from a worldwide sampling of indigenous chickens and their wild counterparts. Our analyses suggest that domestic chickens share a nearly identical demographic history with their direct wild ancestor, the GGS, before the Holocene. Around 12 kya, domestic chickens and GGS have diverged from each other, which is generally consistent with that assessed previously using MSMC based on 50% relative cross-coalescence rate (~9500 ± 3300 years ago) [27]. Subsequently, since domestication, domestic chickens experienced a decline in Ne that was 2.6× more severe than GGS, followed by a recovery towards population expansion. Yet, GGS evolved in a relatively different pattern within this period. We note that the divergence time between domestic chickens and GGS estimated using genomic data is slightly older than the archaeological recordings of chicken domestication; however, the estimated timing of the Ne reduction in domestic chickens generally corresponds with the hypothesized time frame of their domestication [8, 63, 64]. Interestingly, the patterns of Ne differ between domestic chickens and GGS since their split is similar to those observed in both African rice [65] and grape [61] from their wild progenitors, suggesting similar patterns of population size reduction and post-domestication introgression when domesticates spread into new ranges. Our results reveal that the chicken domestication process also followed the same model as what is observed in domestic animals like dogs [66] and horses [12], being initiated with a bottleneck and tailed by a recovery towards population expansion.

Our work reveals that domestic chickens carried 2.95% more hSNPs than GGS, a value comparable to those identified in dogs (2.6%) [13] and rice (∼3–4%) [14], but less than that in grape (~5.2%) [61]. Domestic chickens also held a higher ratio of deleterious to synonymous SNPs and a higher frequency of hSNPs compared with GGS. It is possible that the profound historical Ne decline present in all *Gallus gallus* beginning ~80 kya may have induced the accumulation of hSNPs across all lineages at a comparable magnitude, but the domestication bottleneck and/or recent selection for genetic improvement dramatically increased the genetic load of domestic chickens. A similar pattern has already been observed in horses [67] and dogs [13]. Our results support the “cost of domestication” hypothesis but challenge the proposal of no genetic load during chicken domestication [8, 19, 25, 68]; nonetheless, we could not completely rule out the potential effects from recent genetic improvement and introgression with other jungle fowls on this pattern.

We show that heterozygous hSNPs are accumulated more frequently in the genomes of domestic chickens than GGS, while homozygous ones display a contrary pattern. This is not unexpected, because most harmful mutations are at least partially recessive and therefore could only expose their damaging effects in homozygous states [3]. Especially during breeding practices, harmful mutations in homozygous states are easily observed phenotypically, which promotes purging and breeding decisions, whereas such damaging alleles are masked in heterozygous states and thereby their transmission and accumulation would be facilitated. These results reveal the limitation of current breeding programs in effectively removing potentially damaging effects from the hSNPs while pursuing desirable economic traits. Our study highlights the importance of utilizing genomic information to safeguard genetic improvement through minimizing potential damaging mutations while effectively and sustainably utilizing this species for the poultry industry and biomedical research.

There are several potential caveats in this study. First, our 1K CGP initially aimed to infer the domestication history and evolution of chicken; there is a sampling bias in our study. Our sampling efforts initially focused on diverse and village chickens (which likely present as more “ancient” populations) from Asia (where RJF inhabited) and adjacent regions, while lacking samples from Africa, Oceania, and South America. Even though domestication bottleneck and increased hSNPs are observed in several chicken populations compared with their wild relatives, our samples cannot present the whole genetic diversity of all chickens across the world, and issues on the strength of bottleneck and the number and frequency of these hSNPs accumulated during the early domestication or recent genetic improvement of specific breeds could not be resolved based on our data. Also, there is pervasive gene flow between chickens and other jungle fowls, which also likely result in bias in our estimation of genetic load. Therefore, the magnitudes of bottleneck and genetic load underlying chicken domestication remain open; our analysis provides a result for further testing. Future work by exploring genomes from more heritage chicken lineages and breeds across the world and ancient samples spanning a wide range of periods are necessary to address these questions [67, 69–71]. Second, *TSHR*-559Arg homozygous mice displayed a significant difference in metabolism and development compared with the wild-type mice, suggesting that this mutation is biologically functional. This supports the early study that investigated the function of this mutation based on birds intercrossed between the ancestral RJF (wild type) and White Leghorn [72]. Because of the profound divergence and potential genetic background difference between chickens and mice, we are not able to directly link any phenotypic changes in mice carrying the chicken allele to domestic chickens and RJFs. Our transgenic experiment provides a preliminary biological indicator to unlock *TSHR* function; however, whether it follows the same biological process in both chickens and mice remains unjustified. Third, despite the pattern of hSNPs in domestic chickens is generally consistent with the observations in other species, our genome coverages are relatively low for both domestic chickens and RJFs, and the levels of heterozygotes and het-hSNPs are likely underestimated. Further validation using higher coverage genomes is warranted. Lastly, our analysis focused on variants in the coding regions; however, non-coding regions are increasingly known to play important regulatory roles, and some variants within these regions likely have significant biological functions [73]; future studies should be designed to explore the evolutionary and functional roles of variants within regulatory regions in domestication and genetic improvement of chickens.

## Conclusions

In conclusion, we systematically characterize the existence of a pre-domestication loss of genetic diversity followed by a domestication bottleneck in chickens, leading to the prominence of high-impact alleles across domestic chicken genomes. Through functional trait analyses, we suggest that these high-impact alleles affect behavior, development, and morphology, and our findings indicate that these alleles are partially under artificial selection pressure while the frequencies of detrimental variants are increased due to drift. This study presents a new page in chicken genomics, calling for a sharpened focus on the comparative genomic diversity of specific breeds and wild lineages, and for intensive functional analyses of high-impact alleles, to understand which contribute to domestication and genetic improvement of particular traits and which are maladaptive. This would enable the development of reliable markers for monitoring the concrete impact of genetic improvement and the purging of deleterious mutations on chicken genome evolution. In addition, our dating of the bottleneck and recovery processes in one of the most heavily relied upon domesticated species in the world has broad implications for understanding the biocultural interactions, translocation, and domestication practices affecting suites of species in Eurasia that were exploited in the past. Our study provides a possibility for further investigation using breeding experiments and a larger scale of genomes covering a wider sampling of global chickens and fossils.

## Methods

### Genomic datasets

In our 1K Chicken Genome Project (CGP), we leveraged the Illumina sequencing platform and generated 787 genomes from indigenous chickens and jungle fowls [27]. These samples included domestic chickens (*n* = 620) and all five red jungle fowl subspecies (*G. g. bankiva*, *n* = 3; *G. g. gallus*, *n* = 6; *G. g. murghi*, *n* = 68; *G. g. jabouillei*, *n* = 27; and *G. g. spadiceus*, *n* = 45), as well as green jungle fowls (*G. varius*; *n* = 12), Ceylon jungle fowls (*G. lafayettei*; *n* = 4), and gray jungle fowls (*G. sonnerati*; *n* = 2). Specifically, *G. g. spadiceus* was sampled from Thailand and Yunnan province of China, and domestic chickens were sampled from villages in Indonesia, Thailand, Vietnam, China, India, Sri Lanka, Bangladesh, Pakistan, Iran, Afghanistan, and Europe. By combining an additional 76 published genomes [44, 56, 57, 74–77] and applying the standard BWA-GATK pipeline [78, 79], 33.4 M SNPs were successfully genotyped for 863 birds, of which ~25 million SNPs were identified in domestic chickens (*n* = 696) and *G. g. spadiceus* (*n* = 45). Genotypes for 9 *G. g. spadiceus* samples (ypt2887–ypt2895) from Thailand and 1 *G. g. spadiceus* sample from Daweishan (ypt570) were admixed with chicken [27] and were removed. This resulting dataset (including 696 domestic chickens and 35 *G. g. spadiceus* samples) was used to perform the genetic diversity and genetic load analyses. The dataset is available at ChickenSD (http://bigd.big.ac.cn/chickensd/; released).

Pair-wise sequential Markovian coalescent (PSMC) [38] and multiple sequential Markovian coalescent (MSMC) analyses require high-coverage genomes for the successful calling of genome-wide heterozygosity. We selected high-coverage genomes from previous studies (find detail in Additional file 1: Table S1) [35, 40, 41, 74], including genomes for GGS and 18 diverse chicken populations (Yunnan chicken, Yunnan game fowl, Emei chicken, Muchuan chicken, Hetian chicken, Tulufan chicken, Lindian chicken, Liyang chicken, Xianju chicken, Baier Yellow chicken, Yunyang Da chicken, Laos chicken, Sri Lankan chicken, Ethiopian chicken, and four European commercial chicken breeds (White Recessive Rocks, Cobb chicken, White Leghorn, and Rhode Island Red)). All reads from these samples were mapped to the chicken reference genome (GRCg6a: https://www.ncbi.nlm.nih.gov/assembly/?term=GCA_000002315.5) using the standard BWA-GATK pipeline [78, 79]. Sequencing coverages for these genomes were calculated using samtools with the “depth” function [80].

### Demographic history inferences

PSMC [38], MSMC [44], and SMC++ [45] were used to estimate the effective population size (Ne) changes in domestic chickens and GGS in the past. For PSMC analysis, consensus sequences of each of the individuals were called using samtools with the “mpileup” command (version: 1.3.1; http://samtools.sourceforge.net/). The loci with less than 1/3 or more than 2 times average read depths were deleted, and sites with consensus qualities below 20 were also removed. PSMC was running with parameters set as “psmc -N25 -t15 -r5 -p 4+25*2+4+6”. Input data for MSMC was prepared using the tool generate_multihetsep.py suggested by the author from https://github.com/stschiff/msmc-tools. The genotypes for all samples were phased jointly using Beagle V4.1 [81] with default parameters. For each group, two individuals (four haplotypes) were analyzed.

For running SMC++ (v1.15.2), we sequenced genomes for each of the three GGS samples (IDs ypt3001, ypt3006, and ypt3009) reported [27] previously to coverage over 20-folds. We performed analysis for the regions with reads mapped uniquely that were generated using the SNPable toolkit (http://lh3lh3.users.sourceforge.net/snpable.shtml) with settings “- k=35 and r=0.9”. To maximize more populations to be analyzed, five genomes for each population with coverage over 15-folds were used. Input file for SMC++ was generated using the pipeline as the author suggested (https://github.com/popgenmethods/smcpp). Except that Laos chicken, Emei chicken, and Muchuan chicken have less than five genomes with sequencing depth over 15-folds, we analyzed all populations that were analyzed by MSMC and PSMC above. We further included Jingyang chicken and Pengxia chicken for the SMC++ analysis. SMC++ was ran using default parameters.

Finally, we investigated the population histories by analyzing the joint allele frequency spectra using diffusion approximation for demographic inference (*dadi*) [48]. Because we were mostly interested in the joint demographic history of domestic chickens and GGS, we selected a total of 40 genomes (20 for each group; IDs for GGS: 18833, 19912, Ypt570, Xcelris_174, ypt3003, ypt2893_L3_I025, Xcelris_176, ypt3047, ypt2895_L3_I026, ypt2889, ypt3008, ypt3051, ypt3007, ypt3038, ypt3069, ypt3006, ypt3042, ypt3002, ypt2894, and ypt2887; IDs for domestic chickens: YPt648, Ypt638, 43S, Ypt646, ypt3180, ypt2656, 95S_L8_I025, Ypt606, 19S_L4_I026, 87S_L5_I010, ypt948_L2_I034, Ypt645, 77S_L4_I029, ypt910_L3_I005, 88S_L4_I011, 44S_L4_I001, 130S_L4_I044, ypt907_L6_I002, 39S_L6_I053, and 21S_L6_I028) from the 1K CGP. To avoid evolutionary restrictions as much as possible, we excluded coding regions. We also masked the repeated (annotations from NCBI: https://www.ncbi.nlm.nih.gov/) and low-complexity regions identified using mdust [78]. Finally, 47,307 autosomal regions of at least 1 kb spanning a total of 70,919,324 bp were used for the demographic analysis. We computed two-dimensional site frequency spectra using ANGSD [82], as described previously [70]. We examined four demographic models (Additional file 1: Figure S3): (A) constant without gene flow, (B) constant with asymmetric gene flow, (C) constant-growth/reduction with asymmetric migrations, and (D) constant-growth/reduction with asymmetric migrations. For each model, we ran three sets of increasingly focused optimizations before performing the final model selection. Models were compared using the Akaike Information Criterion (AIC), and the replicate with the highest likelihood for each model was used to calculate AIC and deltAIC. To calculate the confidence interval for the parameters in our best-fitting model, we applied non-parametric bootstrapping (100 replicates).

Estimations from PSMC, MSMC, SMC++, and *dadi* were scaled using a generation time (g) of 1 year and a mutation rate (μ) of 1.91 × 10^−9^ substitutions per site per year [42]. We used VCFtools [83] to average the population-based nucleotide diversity (π) [84] in domestic chickens and GGS (“--window-pi 50000 --window-pi-step 25000”).

### Identification of high-impact mutations and assessment of genetic loads

To examine the evolution of hSNPs in chickens before and after their domestication, we followed a similar pipeline as described previously [60, 61] to analyze the 33.4 M SNPs called from the 863 genomes. First, we retrieved non-synonymous mutations as annotated by ANNOVAR [85] and searched the chicken genome annotations from the ENSEMBL database (version 83: http://dec2015.archive.ensembl.org/index.html). The ancestral state of a variant was inferred from the green jungle fowl. To predict the effect of a missense mutation on a protein, we applied the PROVEAN software [50] in searching the non-redundant protein database (download from NCBI: https://www.ncbi.nlm.nih.gov/). The prediction was based on evolutionary conservation by comparing the query and target sequences. Similar to the early study [14], a mutation with a PROVEAN score less than −2.5 was considered to be harmful, and such kinds of variants were labeled as hSNPs.

To obtain a global perspective on the functions of genes carrying such hSNPs, we used g:Profiler [86] to retrieve the functional enrichment terms, including Gene Ontology (GO, KEGG pathways) and Human Phenotype Ontologies (HPOs). To assess the landscape of genetic loads over chicken domestication, we calculated the number and frequency of hSNPs per individual or region and compared them with those of synonymous mutations.

Furthermore, we retrieved genomic regions of putatively selective sweeps that were measured by locus-specific branch length (LSBL) statistics [87] in the combination of LSBL1 (chicken; *G. g. spadiceus*, *G. g. jabouillei*) and LSBL2 (chicken; *G. g. spadiceus*, *G. g. murghi*), as well as π-ratio (π_*G.g.spadiceus*_/π_chicken_) [84] from our previous study [27]. For sweep regions identified by each of the three statistics, we compared the number and frequency of hSNPs within the sweeps to the remaining genomic regions between domestic chickens and GGS.

### Construction of *TSHR* knock-in mice

The allele *TSHR*-Gly558 (chr5:40,089,599G) in domestic chickens is highly conserved across vertebrates and corresponds to the mice-*TSHR*-Gly559 (c.1675G) in the 10th exon of transcript Tshr-202 (http://www.ensembl.org/Mus_musculus/Transcript/Exons?db=core;g=ENSMUSG00000020963;r=12:91400994-91540509;t=ENSMUST00000021346). A C57BL/6 mouse model with a mutation at the mouse *TSHR* locus (p. Gly559Arg; c.1675G>A) was constructed by CRISPR/Cas-mediated genome engineering (Shanghai Biomodel Organism Science & Technology Development Co., Ltd). Briefly, Cas9 mRNA, gRNA, and donor DNA were micro-injected into the fertilized eggs of C57BL/6J mice to obtain F0 generation mice with the mutation of the target site (Additional file 1: Figure S5 and Table S6). The F0 generation mice were mated with C57BL/6J mice to obtain positive and homozygous F1 generation mice. All mice had free access to food and water. All experiments were performed following the Health Guide for the Care and Use of Laboratory Animals and were approved by the Ethics Committee of the Kunming Institute of Zoology, CAS.

### Metabolism assay of the transgenic mice

A Comprehensive Laboratory Animal Monitoring System (CLAMS) was used to monitor and analyze the indexes of metabolism and feed intake of the transgenic mice. Eight-week-old male mice were weighed (*n* = 8*2) and placed in CLAMS (PRO-MRR-8) to measure the metabolism of homozygous (HO) and wild-type mice at 30 °C, 18 °C, and 5 °C for 72 h. The levels of oxygen consumption (VO_2_), carbon dioxide exhalation (VCO_2_), calorie consumption, and water and food intake were recorded every 5 min for each mice (including HO and wild type). All mice had free access to water and food and were subjected to the same day-night cycle during the examination. Statistical significance was measured by Student’s test (two-tailed), and *P* < 0.05 was accepted to be significant.

## Supplementary Information

**Additional file 1: Figure S1.** Nucleotide diversity for *G. g. spadiceus* and chicken populations (grouped by samling locations and breeds). **Figure S2.** Demographic histories for *G. g. spadiceus* and diverse chicken groups by PSMC. A total of 18 chicken populations were included in this analysis. **Figure S3.** Four tested demographic models for *dadi* analysis. Nanc, ancestral population size before the split; T, timepoints; m, migrations; Napop, ancestral population size after split. Ncpop, current population size. Arrows depict migration directions. **Figure S4.** Comparing observed data and model allele frequency spectrum for the best model (Model 3). **Figure S5.** Provean-scores for nonsynonymous mutations (for all mutations, left; and for mutations with Provean-scores ≤ –2.5, right) in microchromosomes, macrochromosomes, and intermediate chromosomes. **Figure S6.** Pipeline for constructing the mouse model with a mutation at the mouse *TSHR* locus (p. Gly559Arg; c.1675G>A). **Figure S7.** Photograph showing *TSHR*-559Arg knock-in homozygous (HO) and wild-type mice at 10 months old. **Figure S8.** Gly558Arg knock-in mice consumed less food than wild-type. *, *P* < 0.05. Statistical significance was measured by the Student’s t test. N = 8 for both HO and wild-type male mice were used in each test. **Figure S9.** Number and ratio of high-impact mutations among chicken populations. GGS, *G. g. spadiceus*; DC, all domestic chickens; WL, White Leghorn; TC, Tibetan chicken, XJ, Xinjiang local chicken; You; Beijing You chicken. **Figure S10.** Number and frequency of deleterious mutations in the genomic regions of putatively selective sweeps. **Table S1.** Information for high-coverage genomes used for PSMC, MSMC, and SMC++ analyses. **Table S2.** Estimations of likelihoods and AIC scores from four demographic models. **Table S3.** Summary of population histories calculated from 2D–SFS. Confidence intervals (95%) were obtained by bootstrapping all sites and performing parameter inference on each bootstrap dataset with 100 runs. **Table S4.** Distribution of variants identified in dog, sheep, goat, cattle, pig, and horse. This data is from our previous publication. **Table S5.** GO enrichment for genes carrying nonsynonymous mutation with provean-score of <–10. **Table S6.** Guide RNA sequences for the exon 10 of mouse-*TSHR*.

## Data Availability

All data generated or analyzed during this study are included in this published article and its supplementary information files. Information for the published dataset for estimating demographic histories is available in Additional file 1: Table S1. The genotype data we analyzed (in VCF format) and three GGS genomes we generated were available at ChickenSD (http://bigd.big.ac.cn/chickensd/).

## Abbreviations

RJF: Red jungle fowl
GGS: *G. g. spadiceus*
Ne: Effective population size
1K CGP: 1K Chicken Genomes Project
hSNPs: High-impact SNPs
PSMC: Pair-wise sequential Markovian coalescent
MSMC: Multiple sequential Markovian coalescent
GO: Gene Ontology
HPO: Human Phenotype Ontology
LSBL: Locus-specific branch length
Kya: Thousand years ago
Mya: Million years ago
AIC: Akaike information criterion
HO: Homozygous
WT: Wild-type
