# Supplementary material for: Large-scale genomic analysis reveals the genetic cost of chicken domestication
Source: BMC Biol. 2021 Jun 16;19:118. doi: 10.1186/s12915-021-01052-x (PMC8207802; doi:10.1186/s12915-021-01052-x)
Supplement: Supplementary file 1 — Additional file 1: Figure S1. Nucleotide diversity for G. g. spadiceus and chicken populations (grouped by samling locations and breeds). Figure S2. Demographic histories for G. g. spadiceus and diverse chicken groups by PSMC. A total of 18 chicken populations were included in this analysis. Figure S3. Four tested demographic models for dadi analysis. Nanc, ancestral population size before the split; T, timepoints; m, migrations; Napop, ancestral population size after split. Ncpop, current population size. Arrows depict migration directions. Figure S4. Comparing observed data and model allele frequency spectrum for the best model (Model 3). Figure S5. Provean-scores for nonsynonymous mutations (for all mutations, left; and for mutations with Provean-scores ≤ –2.5, right) in microchromosomes, macrochromosomes, and intermediate chromosomes. Figure S6. Pipeline for constructing the mouse model with a mutation at the mouse TSHR locus (p. Gly559Arg; c.1675G>A). Figure S7. Photograph showing TSHR-559Arg knock-in homozygous (HO) and wild-type mice at 10 months old. Figure S8. Gly558Arg knock-in mice consumed less food than wild-type. *, P < 0.05. Statistical significance was measured by the Student’s t test. N = 8 for both HO and wild-type male mice were used in each test. Figure S9. Number and ratio of high-impact mutations among chicken populations. GGS, G. g. spadiceus; DC, all domestic chickens; WL, White Leghorn; TC, Tibetan chicken, XJ, Xinjiang local chicken; You; Beijing You chicken. Figure S10. Number and frequency of deleterious mutations in the genomic regions of putatively selective sweeps. Table S1. Information for high-coverage genomes used for PSMC, MSMC, and SMC++ analyses. Table S2. Estimations of likelihoods and AIC scores from four demographic models. Table S3. Summary of population histories calculated from 2D–SFS. Confidence intervals (95%) were obtained by bootstrapping all sites and performing parameter inference on each bootstrap dataset with 100 ru [file 12915_2021_1052_MOESM1_ESM.pdf]

Additional file 1:

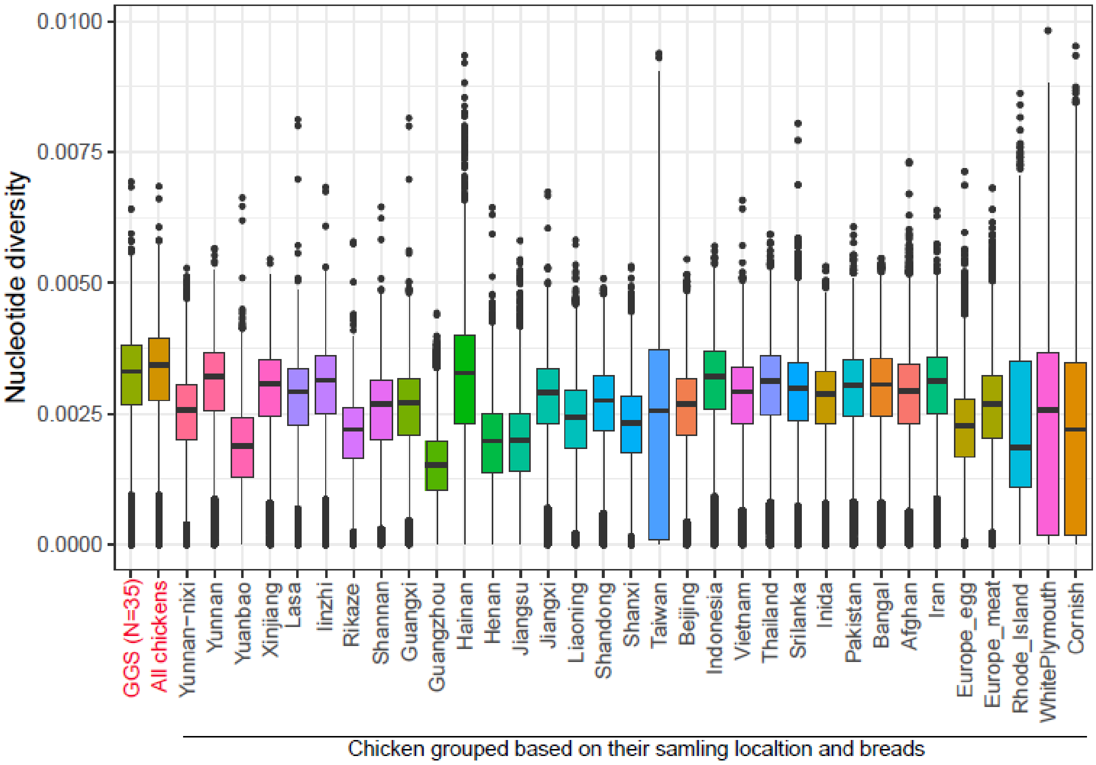

Figure S1: Nucleotide diversity for *G. g. spadiceus* and chicken populations (grouped by sampling locations and breeds).

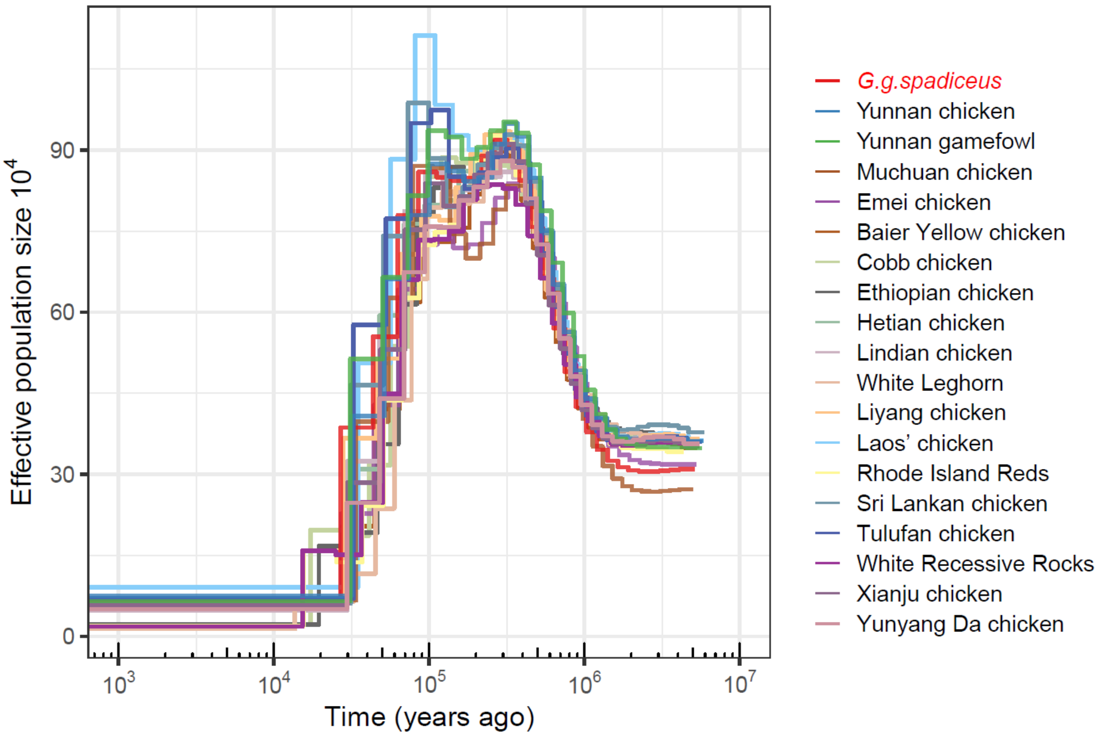

Figure S2: Demographic histories for *G. g. spadiceus* and diverse chicken groups by PSMC. A total of 18 chicken populations were included in this analysis.

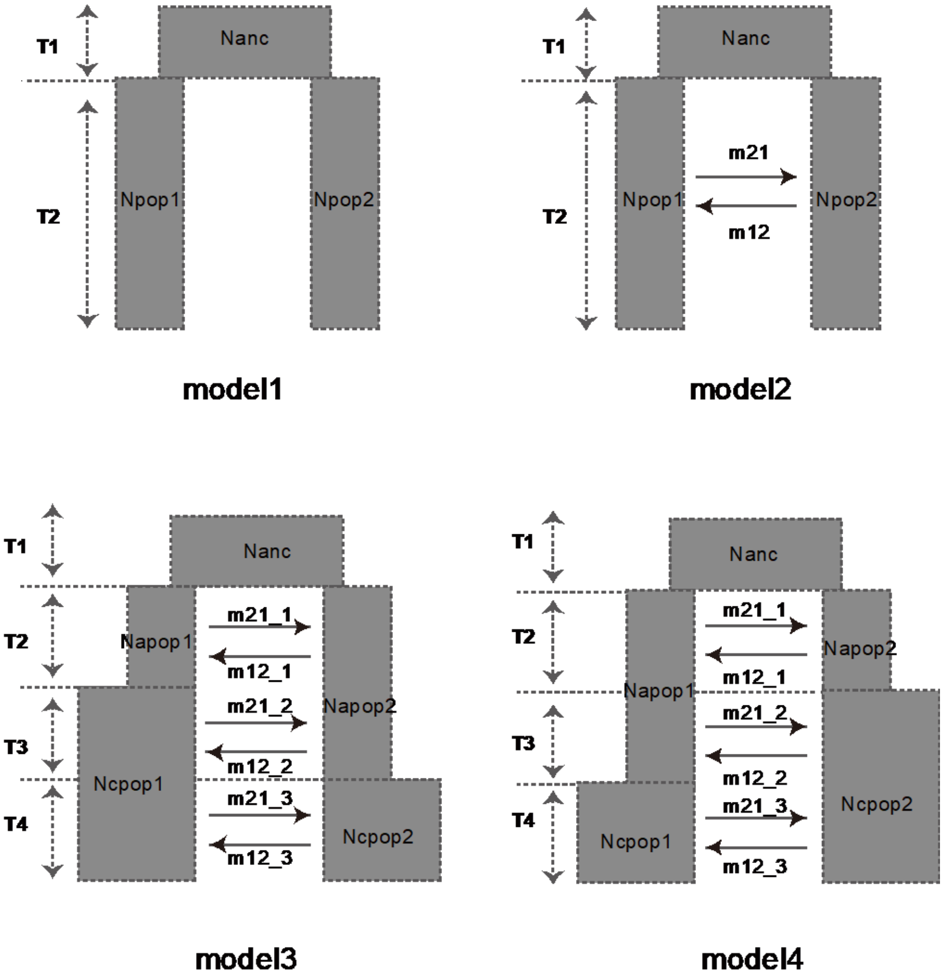

Figure S3: Four tested demographic models for dadi analysis. Nanc, ancestral population size before the split; T, timepoints; m, migrations; Napop, ancestral population size after split. Ncpop, current population size. Arrows depict migration directions.

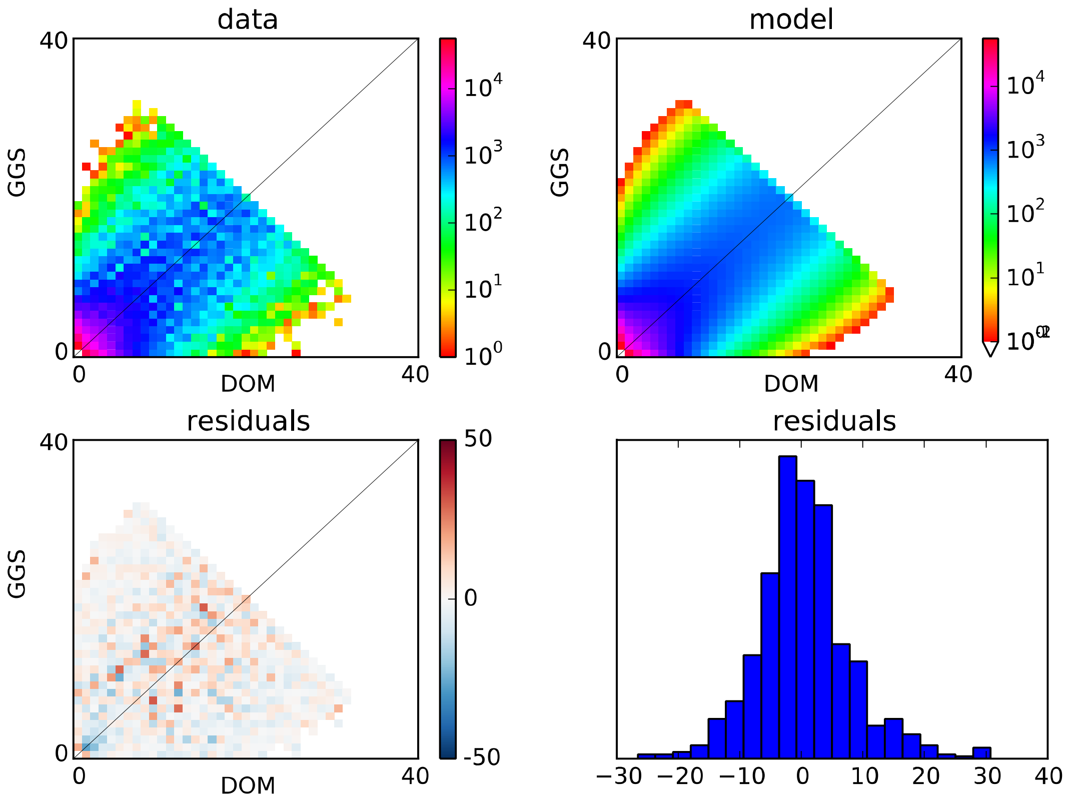

Figure S4: Comparing observed data and model allele frequency spectrum for the best model (Model 3).

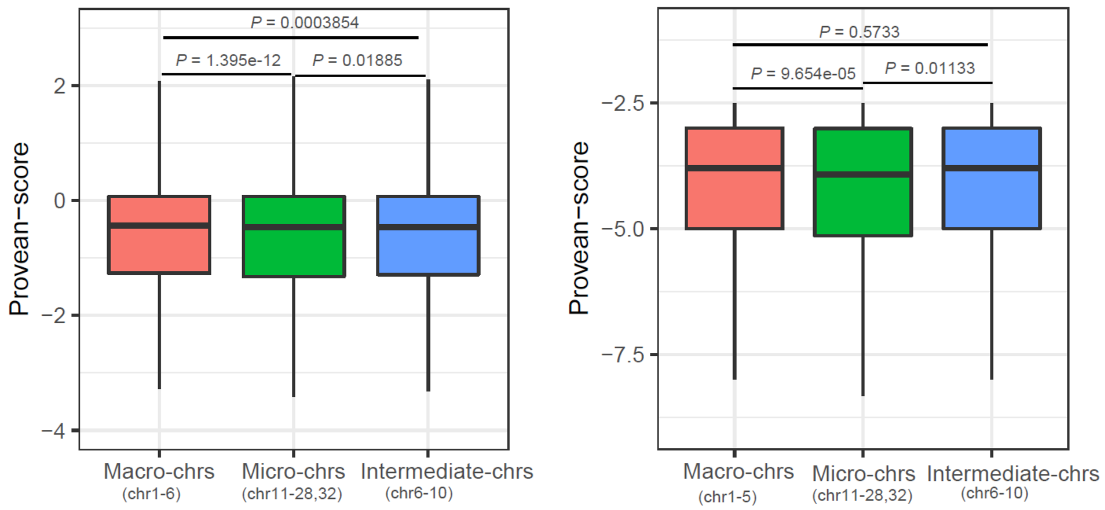

Figure S5: Provean-scores for nonsynonymous mutations (all, left; and for mutations with Provean-scores ≤ –2.5, right) in microchromosomes, macrochromosomes, and intermediate chromosomes.

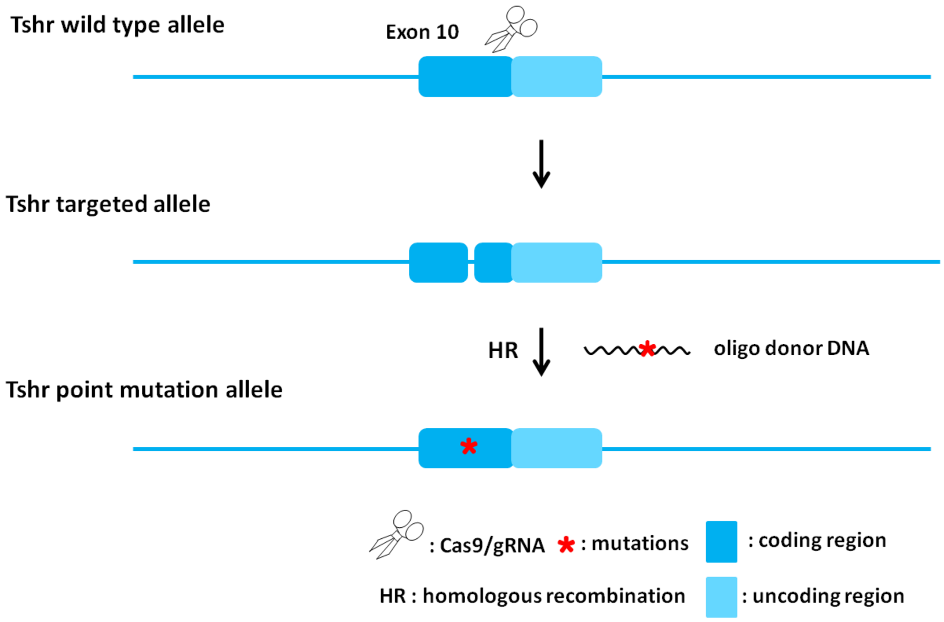

Figure S6: Pipeline for constructing the mouse model with a mutation at the mouse *TSHR* locus (p. Gly559Arg; c.1675G>A).

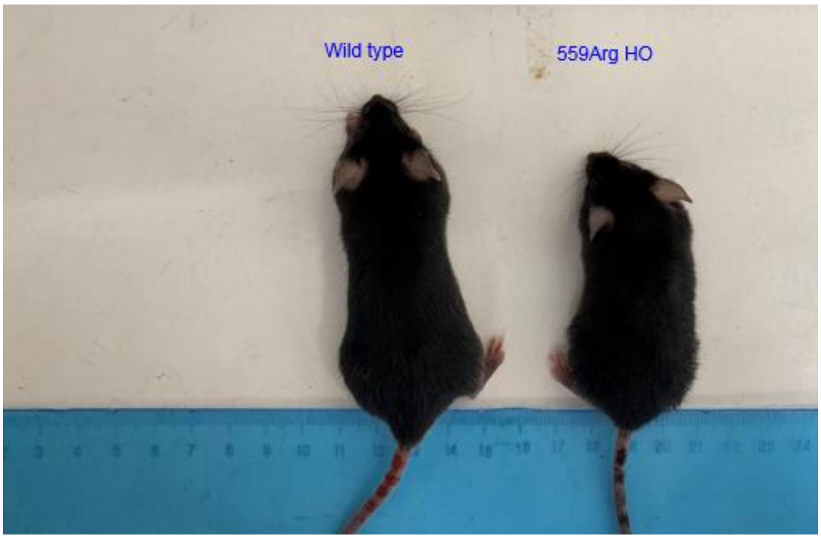

Figure S7: Photograph showing *TSHR*-559Arg knock-in homozygous (HO) and wild-type mice at 10 months old.

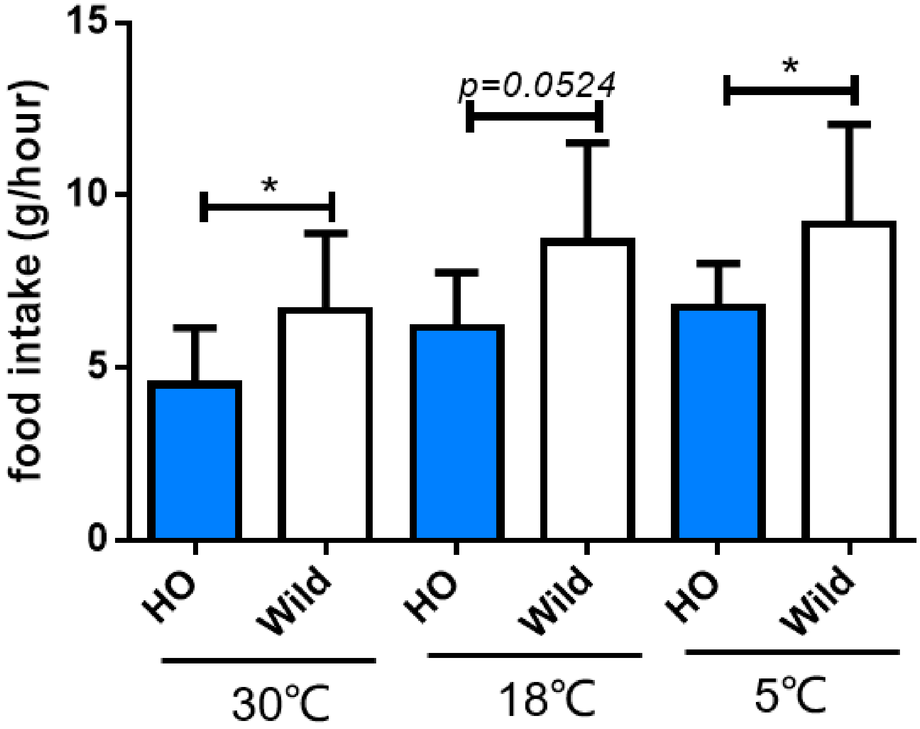

Figure S8: Gly558Arg knock-in mice consumed less food than wild-type. *, $P < 0.05$. Statistical significance was measured by Student's t test. N = 8 for both HO and wild-type mice were used in each test.

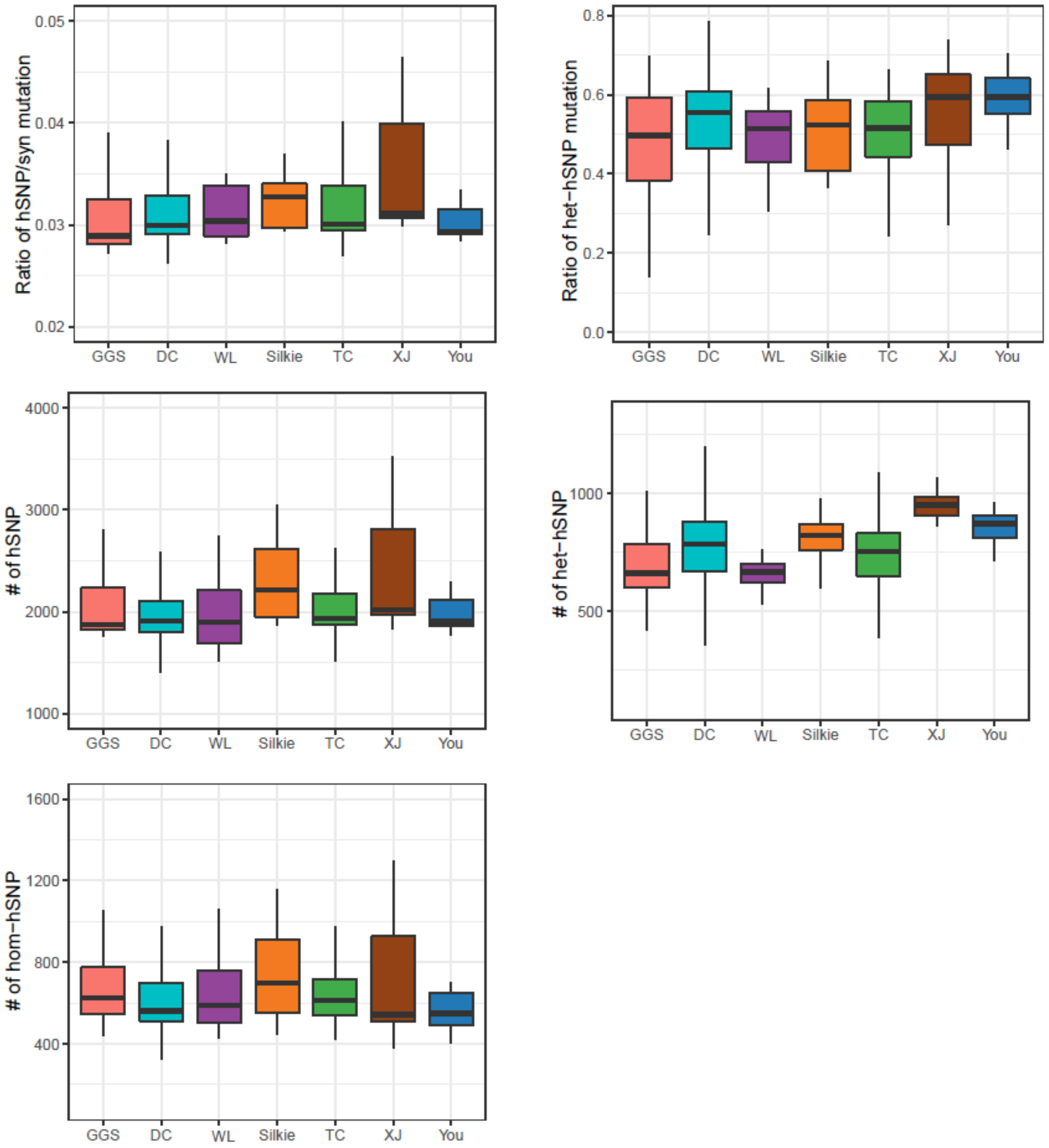

Figure S9: Number and ratio of high-impact mutations among chicken populations. GGS, *G. g. spadiceus*; DC, all domestic chickens; WL, White Leghorn; TC, Tibetan chicken, XJ, Xinjiang local chicken; You; Beijing You chicken.

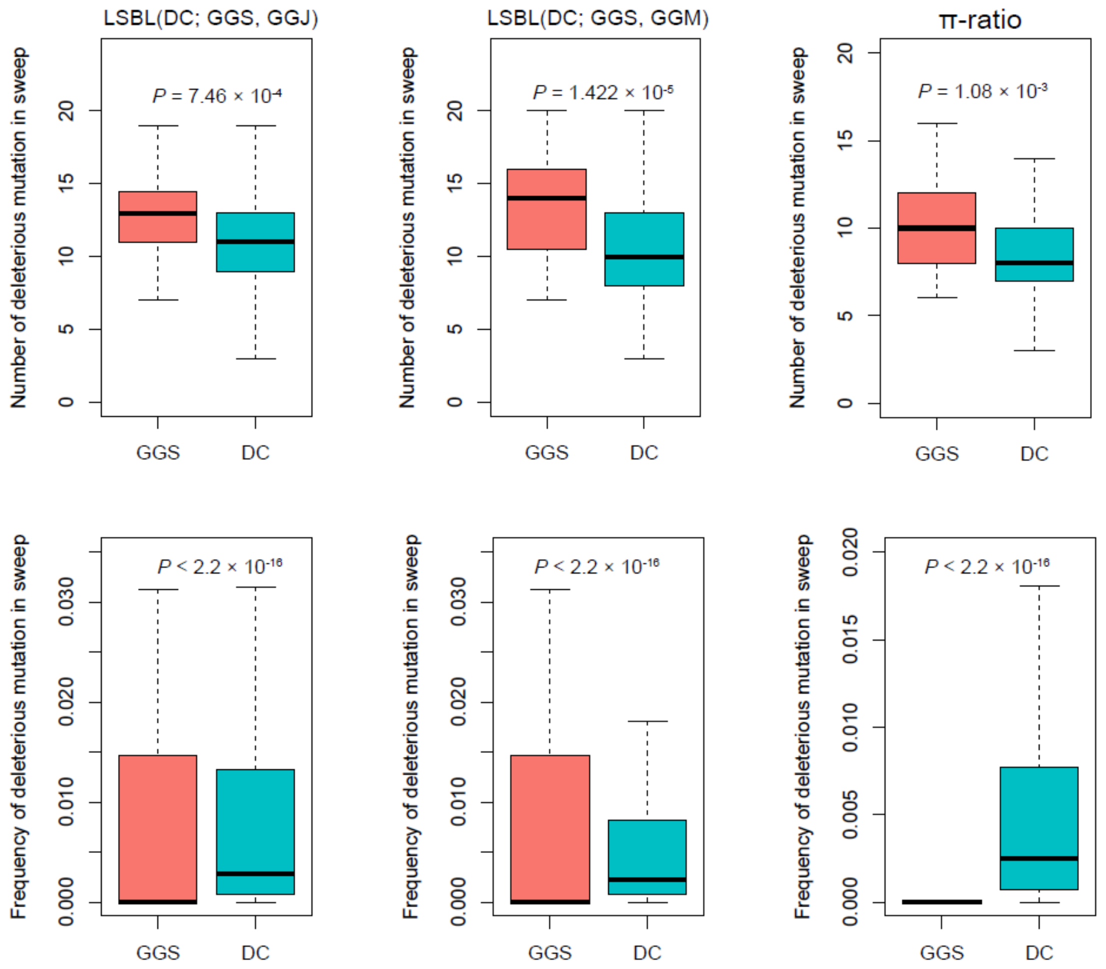

Figure S10: Number and frequency of deleterious mutations in the genomic regions of putatively selective sweeps.

LSBL (DC; GGS, GGM) and LSBL (DC; GGS, GGJ) depict LSBL statistics calculated with domestic chickens as target population and *G. g. murghi* (GGM) or *G. g. jabouillei* (GGJ) as outgroup. GGS, *G. g. spadiceus*.

Table S1: Information for high-coverage genomes used for PSMC, MSMC, and SMC++ analyses.

**IDs**	**Breeds/Groups**	**Sources**
18833	*G. g. spadiceus*	http://bigd.big.ac.cn/chickensd/
19912	*G. g. spadiceus*	http://bigd.big.ac.cn/chickensd/
ypt3001	*G. g. spadiceus*	http://bigd.big.ac.cn/chickensd/
ypt3006	*G. g. spadiceus*	http://bigd.big.ac.cn/chickensd/
ypt3009	*G. g. spadiceus*	http://bigd.big.ac.cn/chickensd/
SRR12145490	Hetian chicken	PRJNA627080
SRR12145489	Hetian chicken	PRJNA627080
SRR12279034	Hetian chicken	PRJNA627080
SRR12279033	Hetian chicken	PRJNA627080
SRR12279032	Hetian chicken	PRJNA627080
SRR3041129	Jingyang chicken	PRJNA306389
SRR3041130	Jingyang chicken	PRJNA306389
SRR3041132	Jingyang chicken	PRJNA306389
SRR3041133	Jingyang chicken	PRJNA306389
SRR3041128	Jingyang chicken	PRJNA306389
SRR12145484	Lindian chicken	PRJNA627080
SRR12145483	Lindian chicken	PRJNA627080
SRR12145482	Lindian chicken	PRJNA627080
SRR12145481	Lindian chicken	PRJNA627080
SRR12145480	Lindian chicken	PRJNA627080
SRR12145478	White Leghorn	PRJNA627080
SRR12145476	White Leghorn	PRJNA627080
SRR12145475	White Leghorn	PRJNA627080
SRR12145474	White Leghorn	PRJNA627080
SRR12145473	White Leghorn	PRJNA627080
SRR12145460	Liyang chicken	PRJNA627080
SRR12145459	Liyang chicken	PRJNA627080
SRR12145458	Liyang chicken	PRJNA627080
SRR12145457	Liyang chicken	PRJNA627080
SRR12145456	Liyang chicken	PRJNA627080
SRR3041413	Pengxian chicken	PRJNA306389
SRR3041414	Pengxian chicken	PRJNA306389
SRR3041415	Pengxian chicken	PRJNA306389
SRR3041417	Pengxian chicken	PRJNA306389
SRR3041418	Pengxian chicken	PRJNA306389
SRR12145453	Rhode Island Red	PRJNA627080
SRR12145452	Rhode Island Red	PRJNA627080
SRR12145451	Rhode Island Red	PRJNA627080
SRR12145450	Rhode Island Red	PRJNA627080

SRR12145449	Rhode Island Red	PRJNA627080
SRR12145441	Cobb chicken	PRJNA627080
SRR12145440	Cobb chicken	PRJNA627080
SRR12145439	Cobb chicken	PRJNA627080
SRR12145438	Cobb chicken	PRJNA627080
SRR12145437	Cobb chicken	PRJNA627080
SRR7062647	Sri Lankan village chicken	PRJNA453469
SRR7062652	Sri Lankan village chicken	PRJNA453469
SRR7062653	Sri Lankan village chicken	PRJNA453469
SRR7062665	Sri Lankan village chicken	PRJNA453469
SRR7062664	Sri Lankan village chicken	PRJNA453469
SRR12145423	Tulufan chicken	PRJNA627080
SRR12145421	Tulufan chicken	PRJNA627080
SRR12145420	Tulufan chicken	PRJNA627080
SRR12145419	Tulufan chicken	PRJNA627080
SRR12145418	Tulufan chicken	PRJNA627080
SRR12145416	White Recessive Rock	PRJNA627080
SRR12145415	White Recessive Rock	PRJNA627080
SRR12145414	White Recessive Rock	PRJNA627080
SRR12145413	White Recessive Rock	PRJNA627080
SRR12145412	White Recessive Rock	PRJNA627080
SRR12145519	Xianju chicken	PRJNA627080
SRR12145518	Xianju chicken	PRJNA627080
SRR12145517	Xianju chicken	PRJNA627080
SRR12145516	Xianju chicken	PRJNA627080
SRR12145515	Xianju chicken	PRJNA627080
Yt527	Yunnan village chicken	http://bigd.big.ac.cn/chickensd/
Yt518	Yunnan village chicken	http://bigd.big.ac.cn/chickensd/
Yt530	Yunnan village chicken	http://bigd.big.ac.cn/chickensd/
Yt529	Yunnan village chicken	http://bigd.big.ac.cn/chickensd/
Yt525	Yunnan village chicken	http://bigd.big.ac.cn/chickensd/
SRR12145502	Yunyang Da chicken	PRJNA627080
SRR12145501	Yunyang Da chicken	PRJNA627080
SRR12145500	Yunyang Da chicken	PRJNA627080
SRR12145499	Yunyang Da chicken	PRJNA627080
SRR12145497	Yunyang Da chicken	PRJNA627080
SRR7062658	Ethiopian village chicken	PRJNA453469
SRR7062659	Ethiopian village chicken	PRJNA453469
SRR7062660	Ethiopian village chicken	PRJNA453469
SRR7062661	Ethiopian village chicken	PRJNA453469
SRR7062662	Ethiopian village chicken	PRJNA453469
Yt519	Game fowl	http://bigd.big.ac.cn/chickensd/
Yt520	Game fowl	http://bigd.big.ac.cn/chickensd/

Yt522	Game fowl	http://bigd.big.ac.cn/chickensd/
Yt524	Game fowl	http://bigd.big.ac.cn/chickensd/
Yt531	Game fowl	http://bigd.big.ac.cn/chickensd/
SRR12145444	Baier Yellow chicken	PRJNA627080
SRR12145433	Baier Yellow chicken	PRJNA627080
SRR12145422	Baier Yellow chicken	PRJNA627080
SRR12145411	Baier Yellow chicken	PRJNA627080
SRR12145520	Baier Yellow chicken	PRJNA627080
SRR3041115	Emei chicken	PRJNA306389
SRR3041121	Emei chicken	PRJNA306389
SRR3041137	Muchuan chicken	PRJNA306389
SRR3041164	Muchuan chicken	PRJNA306389

Table S2: Estimations of likelihoods and AIC scores from four demographic models.

**Models**	**LogLikelihoods**	**Parameters**	**AICs**	**deltaAICs**
Model 1	–23842.15	5	47694.3	4569.7
Model 2	–22944.93	7	45903.86	2779.26
**Model 3**	**–21547.30**	**15**	**43124.6**	**0**
Model 4	–22695.97	15	45421.94	2297.34

AIC = 2*parameters – 2*LogLikelihood; deltaAIC = AIC – min (AIC).

Table S3: Summary of population histories calculated from 2D-SFS. Confidence intervals (95%) were obtained by bootstrapping all sites and performing parameter inference on each bootstrap dataset with 100 runs.

**Parameters**	**Descriptions**	**Point estimations**	**Lower bounds**	**Upper bounds**
Nanc	Ancestral population size	137774	134869	163864
T1	The scale time before split	2833	2384	2921
Napop1	Size of population 1 after split	256927	232755	274288
Napop2	Size of population 2 after split	51687	51612	60207
Ncpop1	Present size of population 1	154433	132332	158533
Ncpop2	Present size of population 2	152156	120894	156331
T2	The scale time between the split and population 1 size change	2098	1541	2801
T3	The scale time between the population 1 size change and population 2 size change	3289	2429	3777
T4	The scale time between the population 2 size change and the present	6990	6896	8941
m12_1	Migration from population 2 to population 1 during T2	4.62e-7	4.58e-7	5.37e-7
m21_1	Migration from population 1 to population 2 during T2	1.81e-7	1.81e-7	2.12e-7
m12_2	Migration from population 2 to population 1 during T3	1.49e-6	1.53e-6	1.80e-6
m21_2	Migration from population 1 to population 2 during T3	1.46e-6	1.46e-6	1.72e-6
m12_3	Migration from population 2 to population 1 during T4	8.97e-6	1.21e-5	1.35e-5
m21_3	Migration from population 1 to population 2 during T4	1.74e-5	1.72e-5	1.74e-5

Table S4. Distribution of variants identified in dog, sheep, goat, cattle, pig, and horse. This data is from our previous publication [1].

**Species**	**#All SNPs**	**#Exonic SNPs**	**%Exonic SNPs**	**#Non-synonymous SNPs**	**%Non-synonymous/Exonic SNPs**
Dog	26425520	210971	0.80%	94000	44.56%
Sheep	46212627	341838	0.74%	144879	42.38%
Goat	57900707	646258	1.12%	259664	40.18%
Cattle	43699310	305877	0.70%	119520	39.07%
Pig	47550124	284827	0.60%	101253	35.55%
Horse	18313274	139329	0.76%	63850	45.83%

Note:

1. Wu, D.-D. *et al.* Convergent genomic signatures of high-altitude adaptation among domestic mammals. *National Science Review* 2020; 7(6):952-963. https://doi.org/10.1093/nsr/nwz213

Table S5: GO enrichment for genes carrying nonsynonymous mutations with Provean-scores of < –10.

GO IDs	Term IDs	*P* values	Gene numbers	Descriptions
GO:0007568	BP	1.85E–02	2	aging
GO:0007569	BP	9.20E–03	2	cell aging
GO:0001935	BP	1.31E–02	2	endothelial cell proliferation
GO:0001936	BP	8.60E–03	2	regulation of endothelial cell proliferation
GO:0050680	BP	1.92E–02	2	negative regulation of epithelial cell proliferation
GO:0001937	BP	2.03E–03	2	negative regulation of endothelial cell proliferation
GO:0061008	BP	1.14E–02	2	hepaticobiliary system development
GO:0001889	BP	1.11E–02	2	liver development
GO:0050953	BP	1.92E–02	2	sensory perception of light stimulus
GO:0007601	BP	1.81E–02	2	visual perception
GO:0007606	BP	1.52E–02	2	sensory perception of chemical stimulus
GO:0007608	BP	6.90E–03	2	sensory perception of smell
GO:0050905	BP	2.11E–02	2	neuromuscular process
GO:0050884	BP	6.04E–04	2	neuromuscular process controlling posture
GO:0050900	BP	4.46E–02	2	leukocyte migration
GO:0051056	BP	4.98E–02	2	regulation of small GTPase mediated signal transduction
GO:0046578	BP	4.24E–02	2	regulation of Ras protein signal transduction
GO:1903708	BP	2.72E–02	2	positive regulation of hemopoiesis
GO:0043648	BP	8.02E–03	2	dicarboxylic acid metabolic process
GO:0042180	BP	2.68E–02	2	cellular ketone metabolic process
GO:0006732	BP	4.85E–02	2	coenzyme metabolic process
GO:0006733	BP	1.62E–02	2	oxidoreduction coenzyme metabolic process
GO:0009108	BP	1.24E–02	2	coenzyme biosynthetic process
GO:1901605	BP	4.76E–02	2	alpha-amino acid metabolic process
GO:0009066	BP	3.32E–03	2	aspartate family amino acid metabolic process
GO:0001505	BP	2.23E–02	2	regulation of neurotransmitter levels
GO:0072331	BP	1.96E–02	2	signal transduction by p53 class mediator
GO:0090305	BP	3.90E–02	2	nucleic acid phosphodiester bond hydrolysis
GO:0071214	BP	4.29E–02	2	cellular response to abiotic stimulus
GO:0009582	BP	1.48E–02	2	detection of abiotic stimulus
GO:0009581	BP	1.48E–02	2	detection of external stimulus
GO:0071478	BP	1.88E–02	2	cellular response to radiation
GO:0071103	BP	3.13E–02	2	DNA conformation change
GO:0006898	BP	4.42E–02	2	receptor-mediated endocytosis
GO:0006310	BP	3.90E–02	2	DNA recombination

GO:1901342	BP	3.77E–02	2	regulation of vasculature development
GO:1901343	BP	7.45E–03	2	negative regulation of vasculature development
GO:2000181	BP	6.90E–03	2	negative regulation of blood vessel morphogenesis
GO:0045765	BP	3.43E–02	2	regulation of angiogenesis
GO:0016525	BP	6.36E–03	2	negative regulation of angiogenesis
GO:0070588	BP	3.64E–02	2	calcium ion transmembrane transport
GO:0031098	BP	1.84E–02	3	stress-activated protein kinase signaling cascade
GO:0007600	BP	4.79E–02	3	sensory perception
GO:0006281	BP	4.85E–02	3	DNA repair
GO:0006302	BP	9.50E–03	3	double-strand break repair
GO:0032147	BP	2.12E–02	3	activation of protein kinase activity
GO:0010876	BP	2.63E–02	3	lipid localization
GO:0006869	BP	1.99E–02	3	lipid transport
GO:1901615	BP	4.63E–02	3	organic hydroxy compound metabolic process
GO:0051186	BP	2.15E–02	3	cofactor metabolic process
GO:0051188	BP	2.84E–03	3	cofactor biosynthetic process
GO:0043900	BP	3.75E–02	3	regulation of multi-organism process
GO:0051606	BP	1.08E–02	3	detection of stimulus
GO:0009314	BP	4.22E–02	3	response to radiation
GO:0009416	BP	2.02E–02	3	response to light stimulus
GO:0051347	BP	3.65E–02	4	positive regulation of transferase activity
GO:0033674	BP	3.03E–02	4	positive regulation of kinase activity
GO:0045860	BP	2.51E–02	4	positive regulation of protein kinase activity
GO:0050877	BP	4.45E–02	5	neurological system process
GO:0000278	BP	4.58E–02	5	mitotic cell cycle
GO:0006974	BP	3.37E–02	5	cellular response to DNA damage stimulus
GO:0042383	CC	9.20E–03	2	sarcolemma
GO:0030315	CC	1.16E–03	2	T-tubule
GO:0031461	CC	3.47E–02	2	cullin-RING ubiquitin ligase complex
GO:0031463	CC	9.20E–03	2	Cul3-RING ubiquitin ligase complex
GO:0030425	CC	4.63E–02	2	dendrite
GO:0043025	CC	4.55E–02	2	neuronal cell body
GO:0043292	CC	3.94E–02	2	contractile fiber
GO:0044449	CC	3.26E–02	2	contractile fiber part
GO:0030016	CC	3.73E–02	2	myofibril
GO:0030017	CC	2.92E–02	2	sarcomere
GO:0044448	CC	1.96E–02	2	cell cortex part
GO:0036477	CC	4.09E–02	3	somatodendritic compartment
GO:0005938	CC	1.37E–02	3	cell cortex
GO:0031224	CC	4.66E–04	40	intrinsic component of membrane
GO:0016021	CC	3.62E–04	40	integral component of membrane

GO:0044425	CC	2.75E–03	42	membrane part
GO:0016020	CC	1.48E–02	49	membrane
GO:0072509	MF	3.51E–02	2	divalent inorganic cation transmembrane transporter activity
GO:0015085	MF	2.35E–02	2	calcium ion transmembrane transporter activity
GO:0005262	MF	1.96E–02	2	calcium channel activity
GO:0042393	MF	2.63E–02	2	histone binding
GO:0005200	MF	2.51E–02	2	structural constituent of cytoskeleton
GO:0016903	MF	2.73E–03	2	oxidoreductase activity, acting on the aldehyde or oxo group of donors
GO:0005319	MF	1.31E–02	2	lipid transporter activity
GO:0004518	MF	3.13E–02	2	nuclease activity
GO:0016810	MF	2.04E–02	2	hydrolase activity, acting on carbon-nitrogen (but not peptide) bonds
GO:0008234	MF	3.13E–02	2	cysteine-type peptidase activity
GO:0008237	MF	4.50E–02	2	metallopeptidase activity
GO:0017171	MF	3.30E–02	2	serine hydrolase activity
GO:0008236	MF	3.26E–02	2	serine-type peptidase activity
GO:0004252	MF	2.31E–02	2	serine-type endopeptidase activity
GO:0000287	MF	4.03E–02	2	magnesium ion binding
GO:0035091	MF	4.93E–02	2	phosphatidylinositol binding
GO:1901981	MF	1.96E–02	2	phosphatidylinositol phosphate binding
GO:1902936	MF	8.02E–03	2	phosphatidylinositol bisphosphate binding
GO:0005546	MF	4.17E–03	2	phosphatidylinositol-4,5-bisphosphate binding
GO:0050662	MF	4.29E–02	2	coenzyme binding
GO:0005539	MF	2.92E–02	2	glycosaminoglycan binding
GO:0008201	MF	1.81E–02	2	heparin binding
GO:0051015	MF	2.31E–02	2	actin filament binding
GO:0038024	MF	1.08E–02	2	cargo receptor activity
GO:0016765	MF	5.10E–03	2	transferase activity, transferring alkyl or aryl (other than methyl) groups
GO:1901681	MF	9.69E–03	3	sulfur compound binding
GO:0004930	MF	4.44E–02	4	G-protein coupled receptor activity
GO:0004674	MF	2.89E–02	4	protein serine/threonine kinase activity
GO:0004175	MF	2.45E–02	4	endopeptidase activity
GO:0008289	MF	4.64E–02	4	lipid binding
GO:0005543	MF	1.46E–02	4	phospholipid binding
GO:0004672	MF	4.58E–02	5	protein kinase activity
GO:0016788	MF	4.79E–02	5	hydrolase activity, acting on ester bonds
GO:0042803	MF	3.11E–02	5	protein homodimerization activity
GO:0016301	MF	5.00E–02	6	kinase activity
GO:0008233	MF	1.35E–02	6	peptidase activity
GO:0070011	MF	1.27E–02	6	peptidase activity, acting on L-amino acid

				peptides
GO:0016772	MF	4.26E–02	7	transferase activity, transferring phosphorus-containing groups
GO:0005509	MF	2.96E–03	8	calcium ion binding
GO:0016787	MF	3.57E–02	15	hydrolase activity
GO:0043168	MF	4.56E–02	16	anion binding
GO:0046872	MF	2.71E–02	20	metal ion binding
GO:0043169	MF	2.13E–02	21	cation binding
GO:0043167	MF	2.65E–02	32	ion binding

Table S6: Guide RNA sequences for the exon 10 of mouse-*TSHR*.

**Exon 10**	CCTCAGGCACGCGTACACCATCATGGCTGGGGGCTGGGTTTCCTG CTTCCTTCTCGCCCTGCTCCCGATGGTG**G**GAATCAGCAGCTATGCC AAGGTCAGCATCTGCCTGCCAATGGACA
**Guide RNA #1**	TCTCGCCCTGCTCCCGATGGTGG
**Donor DNA**	TACACCATCATGGCTGGGGGCTGGGTTTCCTGCTTCCTTCTCGCCC TGCTCCCGATGGTG**A**GAATCAGCAGCTATGCCAAGGTCAGCATCT GCCTGCCAATGGACACCGACACCCCTCTT
